# Supplementary material for: Actin Cytoskeleton Polymerization and Focal Adhesion as Important Factors in the Pathomechanism and Potential Targets of Mucopolysaccharidosis Treatment
Source: Cells. 2023 Jul 5;12(13):1782. doi: 10.3390/cells12131782 (PMC10341097; doi:10.3390/cells12131782)
Supplement: Supplementary file 1 [file cells-12-01782-s001.zip › cells-2425421-supplementary.pdf]

Supplementary Material
for
"Actin Cytoskeleton Polymerization and Focal Adhesion as Important Factors in the Pathomechanism and Potential Targets for Mucopolysaccharidosis Treatment"

Figure S1. F-actin abundance in cells incubated in the presence of genistein or appropriate enzyme. Investigated lines of fibroblasts were incubated for 24 h and stained with CellMask™ Green Actin Tracking Stain. Panel A demonstrate representative micrographs of treated control lines and MPS cells. Panels B and C show quantitative analysis from 100 randomly chosen cells (10,000 events). Error bars represent standard deviation of three independent repetitions of a given experiment. Statistical analysis was performed using one-way ANOVA test. Differences were considered statistically significant relative to the control cell line (*) or untreated MPS I or MPS II (#) when p<0.05.

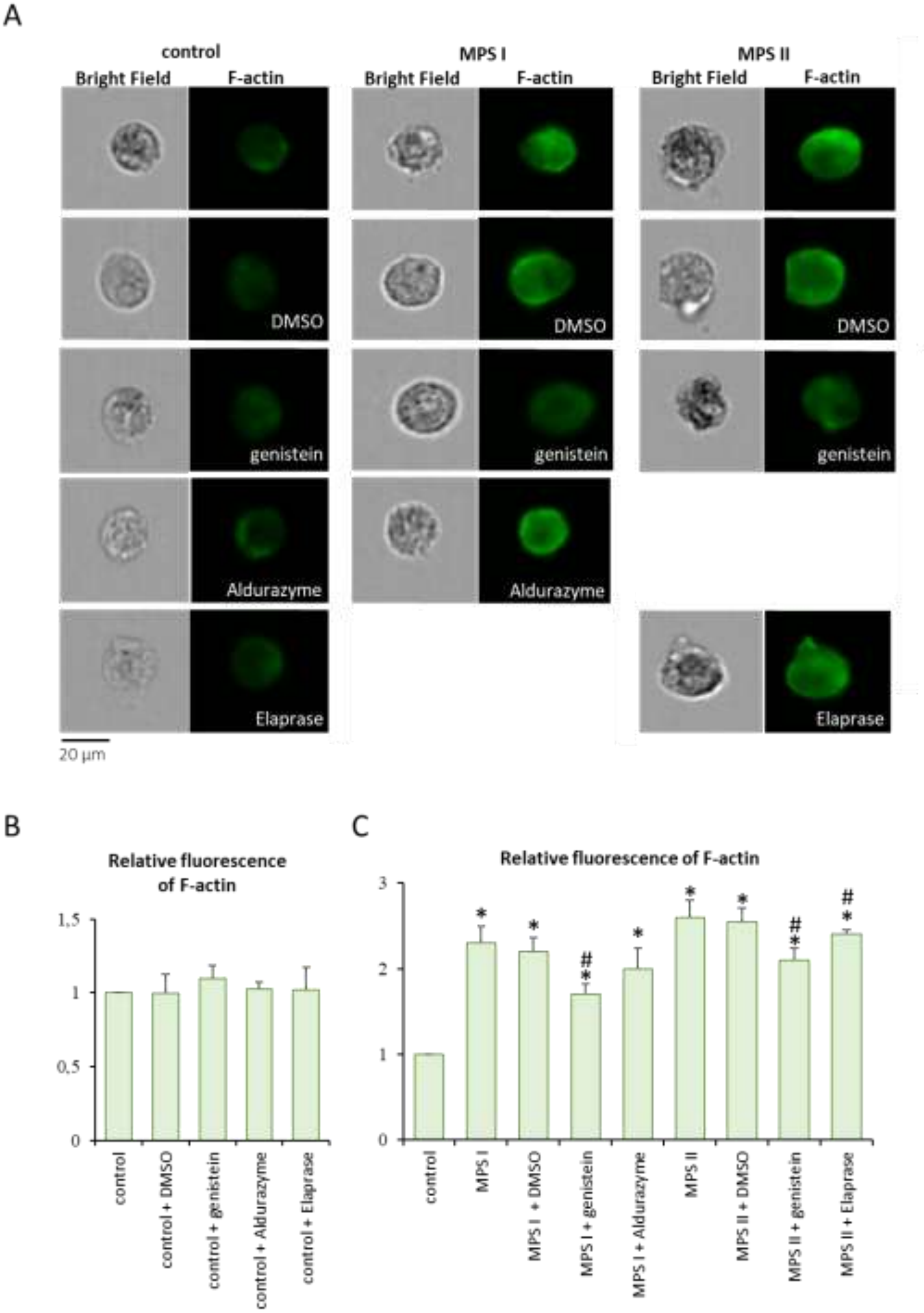

Table S1. Characteristics of MPS patients' derived fibroblast used for transcriptomic analysis.

Cell Line	Sex *	Age *#	Mutated Gene and Its Locus *	Mutation *			
				Allele 1		Allele 2	
				DNA	Protein	DNA	Protein
MPS I	Female	1	*IDUA*, 4p16.3	G1293A	Trp402X	G1293A	Trp402X
MPS II	Male	3	*IDS*, Xp28	208insC	His70ProfsX29	none	none
MPS IIIA	Female	3	*SGSH*, 17q25.3	G1351A	Glu447Lys	G746A	Arg245His
MPS IIIB	Male	7	*NAGLU*, 17q21	C1876T	Arg626X	C1876T	Arg626X
MPS IIIC	Male	8	*HGSNAT*, 8p11.1	ND	ND	ND	ND
MPS IIID	Male	7	*GNS*, 12q14	C1063T	Arg355X	C1063T	Arg355X
MPS IVA	Female	7	*GALNS*, 16q24.3	ND	ND	ND	ND
MPS IVB	Female	4	*GLB1*, 3p22.3	TG851-852CT	Trp273Leu	G1561T	Trp509Cys
MPS VI	Female	3	*ARSB*, 4q14.1	ND	ND	ND	ND
MPS VII	Male	3	*GUSB*, 7q21.11	G1881T	Trp627Cys	G1068A	Arg356X
MPS IX	Female	14	*HYAL1*, 3p.21.3	ND	ND	ND	ND

* According to cell line description in Coriell Institute; #age (years) at the time of cell collection; ND = not determined.

Table S2. Number of up-and down-regulated transcripts (at FDR<0.1;*p*<0.1) of gene included into Actin filament-based process (GO:0030029) term in cells of different MPS type relative to control cells (HDFa).

No. of transcripts with altered expression in particular MPS type vs HDFa line											
**MPS type:**	**I**	**II**	**IIIA**	**IIIB**	**IIIC**	**IIID**	**IVA**	**IVB**	**VI**	**VII**	**IX**
up-regulated	23	13	27	29	21	18	17	22	12	34	18
down-regulated	7	6	13	13	13	3	5	18	4	8	12
**total**	**30**	**19**	**40**	**42**	**34**	**21**	**22**	**48**	**16**	**42**	**30**
